# The relationship between childhood trauma, early maladaptive schema and alexithymia: a multi-group path model of clinical and non-clinical samples

**DOI:** 10.3389/fpsyg.2026.1754917

**Published:** 2026-02-20

**Authors:** Qiuying Zhang, Quandong Liu, Menglu Jia, Sicen Zhang, Lixia Zhang, Zhengtu Cong

**Affiliations:** 1 Hainan Provincial Adolescent Mental Health Medical Center, Hainan Provincial Anning Hospital, Haikou, Hainan, China; 2 Beijing Huilongguan Hospital, Peking University, Beijing, China; 3 Lanxi No. 5 Hospital, Jinhua, Zhejiang, China; 4 Hainan Provincial Institute of Mental Health, Hainan Provincial Anning Hospital, Haikou, Hainan, China

**Keywords:** a multi-group path model, alexithymia, childhood trauma, depression, early maladaptive schema

## Abstract

**Introduction:**

Depression patients with comorbid alexithymia often experience difficulties in emotional expression and emotional recognition. Previous research has identified early maladaptive schemas (EMS) as mediators in the relationship between childhood trauma and alexithymia; however, no studies have examined this mediation in combined models across clinical and non-clinical samples.

**Methods:**

This study included 134 non-clinical participants (Mean age = 37.75, SD = 18.41) and 137 clinical participants diagnosed with depression according to ICD-10 criteria (Mean age = 35.80, SD = 11.22). Participants completed the Childhood Trauma Questionnaire, Young Schema Questionnaire, Beck Depression Inventory, and Toronto Alexithymia Scale. Using multi-group path analysis, we tested a hypothesized model in which EMS mediate the relationship between childhood maltreatment and alexithymia in both clinical and non-clinical samples. Additionally, we examined whether structural paths differed between groups.

**Results:**

Results revealed that the disconnection and rejection, impaired autonomy and performance, other directedness, over-vigilance and inhibition and impaired limits schema domains significantly mediated the relationship in both samples. Notably, the association between the over-vigilance and inhibition schema domain and depression was significantly stronger in the clinical sample compared to the nonclinical sample.

**Discussion:**

These findings highlight the mediating role of EMS in the link between childhood trauma and alexithymia and underscore group-specific differences in schema depression associations, with implications for tailored clinical interventions.

## 1 Introduction

Depression is characterized by persistent low mood, anhedonia, reduced volition, increased fatigue and slowed cognitive processing. Individuals with severe depression may develop suicidal ideation and even behaviors that severely impact quality of life and daily functioning. Depression is a prevalent mental health condition, affecting over 280 million people worldwide as reported by the World Health Organization. In China, the number of individual with depression exceeds 95 million, surpassing the global average and ranking as the second leading cause of disease burden in the country (World Health Organization, 2022). In recent years, the incidence of depression has shown a trend of younger populations, with a lifetime prevalence of 15–20% among adolescents, making it one of the most burdensome psychiatric disorders (American Psychiatric Association, 2013). Despite the availability of advanced therapeutic technologies for depression, there remains a lack of systematic prevention and intervention strategies for this chronic condition. Therefore, exploring novel preventative interventional approaches for a more systematic management of depression is of crucial importance.

The development of a healthy worldview and robust self-concept in children generally requires comprehensive physical and emotional nurturance, opportunities for play and spontaneous exploration, coupled with a secure environment and age-appropriate boundaries (Young et al., 2003). Based on Bowlby and Ainsworth’s attachment theory, the interaction patterns between early caregivers and children are internalized as internal working models. When caregivers consistently fail to meet the child’s fundamental needs, these maladaptive internal representations may disrupt lifelong emotional regulation, interpersonal functioning, and psychological adaptation (Nivison et al., 2021). Early childhood trauma represents one of the most significant risk factors capable of compromising both physiological and emotional integrity in children, and serves as a fundamental etiological contributor to a spectrum of psychopathological outcomes including depression and anxiety (Kuzminskaite et al., 2022). Childhood trauma is typically categorized into five distinct types: sexual abuse, physical abuse, emotional abuse, emotional neglect, and physical neglect. A recent study reported that 59.6% of male participants and 40.0% of female participants had experienced at least one form of childhood trauma. Among these, physical neglect was the most prevalent (44.7%), followed by emotional abuse (40.3%), physical abuse (31.8%), sexual abuse (30.4%), and emotional neglect (30.0%) (Moustafa et al., 2021). Early childhood trauma exerts both short- and long-term impacts across multiple developmental domains (Kendall-Tackett and Marshall, 1998; Moustafa et al., 2021; Yiğit et al., 2021). This type of trauma particularly disrupts the formation of cognitive schemas regarding the self and others (Boyda et al., 2018; Duru and Balkıs, 2022; Wuth et al., 2022). For instance, individuals with a history of witnessing or experiencing violence often exhibit diminished self-esteem in adulthood and display a persistent lack of trust toward others (Branden, 2001). In interpersonal contexts, they tend to interpret others’ intentions as negative and untrustworthy, frequently experiencing heightened fear of intimacy and a marked tendency to avoid forming deep emotional connections (Drapeau and Perry, 2004). Moreover, especially sexually abused children may start to perceive the whole world as a dangerous place. Therefore, it is crucial to understand its outcomes and how it leads to various intra and interpersonal problems. According to the schema theory,

children develop such mental representations, namely Early Maladaptive Schemas (EMS) to understand and interpret their negative experiences (Young et al., 2003). While EMS enables children to maintain vigilance and avoid potential harm in the face of recurring adverse experiences, the prolonged persistence and over-generalization of such anticipatory patterns may lead to the development of maladaptive coping strategies. Consequently, individuals may exhibit unnecessary interpersonal avoidance or aggressive behaviors (Hayes and Parsonnet, 2016; Mojallal et al., 2015; Van Wijk-Herbrink et al., 2021). Concordantly, children who are exposed to traumatic or adverse events, have been found to be at higher risk of depression (Wang et al., 2021), suicide (De Berardis et al., 2018; Orsolini et al., 2020), anxiety, and dissociative disorders (Wildschut et al., 2019). Recent research further supports these pathways, indicating that early maladaptive schemas play a critical role in linking childhood adversity to various forms of psychopathology in vulnerable child populations (Tsouvelas et al., 2023).

In the schema theory, Young et al. (2003) proposed that children who experience early adversity may develop 18 distinct early maladaptive schemas. These schemas are categorized into five different domains based on five core unmet psychological needs: disconnection and rejection (DR), impaired autonomy and performance (IAP), other-directedness (OD), over-vigilance and inhibition (OVI), and impaired limits (IL). Research indicates that children who experience emotional and physical neglect are particularly prone to developing schemas associated with the disconnection and rejection and impaired autonomy and performance domains (Pilkington et al., 2021). According to schema theory, children who experience prolonged deprivation of consistent care and emotional nurturance, along with sustained neglect, abuse, rejection, and humiliation within unstable family environments, develop profound feelings of need frustration. This may lead to the formation of EMS within the disconnection and rejection domain, accompanied by persistent feelings of insecurity, worthlessness, and social isolation (Young et al., 2003). Despite a strong desire for love and attention from others, they often struggle to express their emotions and needs effectively. According to Young et al. (2003), neglected children whose uniqueness, needs, and emotions are consistently ignored or undermined may develop EMS within the impaired autonomy and performance domain. These children often experience pervasive feelings of incompetence and helplessness, and demonstrate a lack of confidence in their own abilities and emotions when navigating daily tasks and responsibilities. Supporting this theoretical framework, research by Pilkington et al. (2021) found that children who experienced emotional and physical neglect were particularly prone to developing schema domains of disconnection and rejection as well as impaired autonomy and performance.

Alexithymia often described as an inability to express emotions,is characterized by three primary dimensions: difficulty recognizing emotions, difficulty expressing emotions and externally oriented thinking. Alexithymia has been recognized as a significant long-term consequence of childhood maltreatment (Güleç et al., 2013; Taylor, 1984). Early childhood trauma is modeled as a distal antecedent, which indirectly contributes to subsequent alexithymia through the disruption of attachment security and neuropsychological development (Zdankiewicz-Ścigała and Ścigała, 2020). Bermond et al. (2008) showed thatpeople who were sexually abused during their childhood obtained higher scores of alexithymia compared to the control group. Consistent with the findings of Zlotnick et al. (2001), severe emotional neglect and physical neglect were significantly associated with elevated levels of alexithymia, with emotional neglect identified as the strongest predictor of alexithymic traits. Thus, childhood trauma impairs the capacity for emotional development and disrupts both biological and psychological processes underlying affect regulation, which may further contribute to the emergence of alexithymia.

Besides, research demonstrate that early maladaptive schemas predict the alexithymia level of individuals. Studies indicate that not only do scores in the Impaired autonomy and performance and over-vigilance and inhibition domains predict alexithymia, but all schema domains are positively correlated with with the alexithymia levels of individuals (Ameri et al., 2014; Abdolmohammadi et al., 2016; Pinna et al., 2020; Mahta and Saeed, 2022). Among these, Saariaho et al. (2015). demonstrated a significant correlation between individuals’ level of alexithymia and scores in the disconnection and rejection schema domain. Furthermore, other studies have identified the Impaired autonomy and performance schema domain as a key predictor of alexithymia; individuals with such schemas tend to hold maladaptive expectations of themselves and their environment, which adversely affect their perceived ability to function independently (Ekinci and Kandemir, 2015). These findings collectively suggest a link between EMS and alexithymia. In the over-vigilance and inhibition schema domain, schemas such as emotional inhibition and unrelenting Standards lead individuals to actively suppress, ignore, or minimize emotional experiences in order to fulfill their needs for control or perfection (Abdolmohammadi et al., 2016). This directly results in difficulties in identifying and describing emotions. However, some studies have refuted the existence of such a relationship, indicating that individuals with EMS do not necessarily develop alexithymia later in life, suggesting that EMS may not directly lead to the onset of alexithymia (van der Velde et al., 2015). These inconsistent findings underscore the need for further research to clarify the relationship between EMS and alexithymia.

As early maladaptive schemas lead individuals to form maladaptive coping strategies including unhealthy emotional states, behaviors, and attitudes (Young et al., 2003), alexithymia might be understood as one of them, which helps individuals to get alienated from painful emotions related to destructive events and relationships. According to survey results, the prevalence of alexithymia in the general population is approximately 10–13% (Terock et al., 2020). The incidence of alexithymia is even higher in clinical populations. The latest research by Ricciardi et al. (2015) found that the prevalence of alexithymia among individuals with mental disorders ranges from 40 to 60%, with anxiety disorders accounting for 13–58%, depressive disorders for 32–51%, eating disorders for 24–77%, and substance abuse disorders for 30–50%. Extensive research has demonstrated a significant association between childhood trauma and alexithymia in adulthood. Notably, this association is not observed in healthy populations, nor is it confined to a single diagnostic category. Instead, it functions as a transdiagnostic pathway prevalent across multiple psychiatric spectra. Specifically, childhood traumatic experiences have been consistently linked to difficulties in emotional identification, description, and externally oriented thinking—the core features of alexithymia—in adult patients. This holds true across mood disorders (e.g., bipolar disorder), anxiety and stress-related disorders, trauma and personality-related disorders, psychotic disorders, as well as in patient groups primarily presenting with somatic symptoms (Takım et al., 2024a; Takım et al., 2024b). This suggests that the progression from childhood trauma to later impairments in emotional expression and identification may represent a common psychopathological mechanism transcending traditional diagnostic boundaries. Therefore, investigating the pathways through which childhood trauma and early maladaptive schemas contribute to alexithymia holds significant theoretical and clinical importance for understanding transdiagnostic vulnerability factors. Understanding the root causes of diseases can aid in developing more effective prevention and intervention strategies for various mental disorders (De Berardis et al., 2017). In summary, existing research primarily has the following limitations: there are relatively few studies on the relationships between childhood trauma, EMS, and alexithymia. Although the detrimental effects of EMS on alexithymia have been confirmed in healthy populations, the mediating role of EMS has not been tested when clinical factors are taken into account.

Comparing the differences in childhood trauma, EMS, and alexithymia between individuals with depression and healthy groups can help therapists conduct a more comprehensive assessment of patients’ childhood trauma, EMS, and alexithymia during disease intervention. When applying schema therapy, therapists can better understand patients’ childhood experiences and pay attention to the manifestations of specific EMS and alexithymia. Studies have found that patients with depression exhibit significant alexithymia. Investigating the relationship between EMS, childhood trauma, and alexithymia helps to understand how schemas and childhood trauma influence alexithymia in patients with depression. This provides new directions for subsequent psychotherapy and intervention, effectively reducing alexithymia levels and alleviating the severity of the disease.

Building upon schema theory and extant research on trauma related psychopathology, we propose an integrated mechanistic model to explain the differential mediating roles of specific EMS domains and their variation across clinical and non-clinical populations. Childhood trauma is conceptualized as a distal vulnerability factor that disrupts normative affective development through two primary pathways: (1) by compromising attachment security and the formation of internal working models of self and others, and (2) by inducing neurobiological and cognitive alterations that hinder emotional awareness and integration. EMS emerge as enduring cognitive-affective representations of these disruptions, serving as proximal mechanisms that organize subsequent emotional processing and regulation strategies. Recent work reinforces that EMS are pivotal in translating early adverse experiences into lasting emotional processing deficits, including alexithymia (Tsouvelas et al., 2023).

The disconnection and rejection domain comprising schemas such as mistrust/abuse and abandonment—is theoretically rooted in interpersonal trauma (e.g., maltreatment, neglect) and perpetuates profound interpersonal alienation and emotional hyper-vigilance. This dynamic may manifest clinically as alexithymia, mediated through the suppression of emotional expression and impaired ability to recognize emotions in social contexts. The over-vigilance and inhibition domain—which includes schemas such as emotional inhibition, unrelenting standards, and pessimism—is conceptualized as reflecting a coping style characterized by cognitive hyper-vigilance and experiential avoidance, core processes underlying depressive and anxiety disorders. Individuals with over-vigilance and inhibition domain may actively suppress or avoid emotional experience in an effort to maintain control or meet perfectionistic demands, thereby directly exacerbating alexithymic traits such as difficulty identifying and describing feelings. Multi-group analysis enabled us to test whether these mechanistic pathways are amplified in clinical depression. We hypothesized stronger indirect effects through the over-vigilance and inhibition and disconnection and rejection schema domains in the clinical group, attributable to their potentially heightened attachment insecurity, elevated comorbid anxiety, and more entrenched patterns of cognitive avoidance and rumination factors that may exacerbate the association between specific schemas and alexithymia (Cano-Lopez et al., 2022). In contrast, other schema domains may represent more generalized vulnerability factors that operate similarly across populations. This framework not only informs our specific hypotheses but also situates the present study within a broader trans-diagnostic perspective on emotion-processing deficits.

Based on theoretical considerations and prior empirical findings, the following hypotheses are proposed:

*H1*: There were significant differences in all factors of childhood trauma, EMS, and alexithymia between the depression group and the healthy adult group.

*H2*: Scores on all factors of childhood trauma, EMS, and alexithymia showed positive correlations.

*H3*: Early maladaptive schemas mediate the relationship between childhood trauma and alexithymia. The five schema domains, including disconnection and rejection, impaired autonomy and performance, other-directedness, over-vigilance and inhibition, and impaired limits, each significantly mediate the relationship between childhood trauma and alexithymia.

*H4*: The structural pathways significantly differ between the clinical group and the control group. The mediating pathways from childhood trauma to alexithymia via the disconnection and rejection and over-vigilance and inhibition schema domains were significantly stronger in the clinical group than in the non-clinical group.

## 2 Methods

### 2.1 Participants

The depression sample was sourced from adult patients with depression attending the outpatient clinics and inpatient departments of Beijing Huilongguan Hospital. This study has been approved by the Ethics Committee of Beijing Huilongguan Hospital. Prior to the commencement of the study, professionally trained investigators informed the participants about the research objectives and procedures, obtained their informed consent, and had them sign informed consent forms. Participants were clinically diagnosed by professional psychiatrists using the ICD-10 criteria. Patients with severe intellectual disabilities or serious organic diseases, those identified by the attending physician as being in an acute phase requiring immediate intervention for acute symptoms (such as suicide risk or delusional states), and individuals with other psychiatric disorders or substance abuse issues were excluded from the study. Patients who met the diagnostic criteria for depression and were in a stable treatment condition were asked to scan a questionnaire QR code on-site to complete the survey. The healthy control group (no diagnosable mental disorder according to ICD-10 criteria) was recruited online. The research purpose was explained in the questionnaire, and informed consent was obtained from all participants.

This study adopted a consecutive sampling method and collected a total of 287 data entries. Among them, 16 cases were excluded due to short questionnaire response times or incomplete entries. The final sample comprised 137 adult depressed patients from the outpatient and inpatient departments of psychiatric hospitals (clinical group) and 134 healthy volunteers (non-clinical group). There were 36 males and 101 females in the study group, with a mean age of 37.75 ± 18.41 years. In the control group, there were 21 males and 113 females, with a mean age of 35.8 ± 11.22 years. Inclusion criteria for the clinical group required a formal diagnosis of depression by a psychiatrist, while the non-clinical group consisted of individuals with no history of psychiatric diagnoses.

### 2.2 Measurements

#### 2.2.1 Childhood trauma questionnaire

To quantify the degree of childhood trauma, we used the Chinese version of the Childhood Trauma Questionnaire (CTQ) translated and revised by Zhao et al. (2005). The scale contains a total of 28 entries and consists of 5 dimensions, including physical abuse, emotional abuse, sexual abuse, physical neglect and emotional neglect. The scale is scored on a 5-point Likert scale ranging from 1 (never) to 5 (always), with higher scores representing more severe traumatic experiences experienced during childhood. There were 6 reverse scoring questions in the questionnaire. The Cronbach’s *α* coefficient for this total scale in this study was 0.89, and the Cronbach’s *α* coefficients for the subscales were 0.77 (emotional abuse), 0.74 (physical abuse), 0.80 (sexual abuse), 0.87 (emotional neglect), and 0.70 (physical neglect). For the total scale, good internal consistency was also observed within the healthy group (*α* = 0.88) and the depression group (*α* = 0.89).

#### 2.2.2 Short version of the Young Schema questionnaire

To measure the early maladaptive schemas (EMS), we used the short version of the Young Schema Questionnaire in Chinese translated and revised by Zhang et al. (2012). The questionnaire contains a total of 75 entries and consists of 5 subscales, including disconnectionand rejection, impaired autonomy and performance, other-directedness, over-vigilance and inhibition, and impaired limits. The questionnaire is scored on a 5-point Likert scale ranging from 1 (not at all me) to 5 (completely me), with higher scores representing higher levels of maladaptive schema and no reverse scoring questions. The questionnaires had a total Cronbach’s *α* coefficient of 0.98, and the Cronbach’s α coefficients for the subscales were 0.96 (DR), 0.94 (IAP), 0.88 (OD), 0.89 (OVI), 0.88 (IL). For the total scale, good internal consistency was also observed within the healthy group (*α* = 0.96) and the depression group (*α* = 0.98).

#### 2.2.3 Toronto alexithymia scale

We used the Chinese version of the Toronto Alexithymia Scale (TAS), which was translated and revised by Jinyao et al. (2003) to quantify the degree of alexithymia. The scale contains a total of 20 entries and consists of 3 subscales, including emotion recognition inability, emotion expression inability, and extraverted thinking. The scale was scored on a 5-point Likert scale. From 1 (strongly disagree) to 5 (strongly agree), higher scores represent higher levels of dysfunctional emotion telling, with 5 reverse scoring questions. The total Cronbach’s α coefficient for this scale in this study was 0.87, and the Cronbach’s α coefficients for the subscales were 0.90 (inability to recognize emotions), 0.75 (inability to express emotions), and 0.85 (extroverted thinking). For the total scale, good internal consistency was also observed within the healthy group (*α* = 0.78) and the depression group (*α* = 0.87).

### 2.3 Statistical analysis

SPSS 26.0 was used for descriptive statistics and correlation analysis, and AMOS 24.0 was employed to establish structural equation models. The skewness and kurtosis of all variables fell within the range of±2, indicating no substantial deviation from the normal distribution (Lumley et al., 2002). The Harman’s single-factor test was employed to assess common method bias. First, the collected data were organized, and outliers were removed according to the inclusion and exclusion criteria, followed by descriptive statistical analysis. Independent samples t-tests were used for between-group comparisons. Pearson correlation analysis was employed to explore the relationships among the variables. Structural equation modeling was constructed to test for mediation effects and conduct multi-group comparative analysis.

## 3 Results

### 3.1 Common method bias analysis

Since this study employed questionnaire and scale surveys, there may be specific common method bias during the research process. To mitigate this issue, anonymous questionnaires were used in this study, and both positively and negatively worded items were included to reduce bias. Additionally, the Harman’s single-factor test was conducted to assess common method bias. The results of Harman’s single-factor test indicated that the first (unrotated) factor accounted for less than 40% of the total variance, suggesting that common method bias is unlikely to be a serious concern in the present data (Fuller et al., 2016). The results of the unrotated factor analysis showed 28 factors with eigenvalues greater than 1, with the first factor accounting for 28.87% of the total variance—below the critical threshold of 40%. This indicates that no significant common method bias was present in this study.

### 3.2 Preliminary analyses

The independent samples t-test was conducted to compare the total scores of childhood trauma (CTQ), early maladaptive schema (EMS) and alexithymia (TAS) between the clinical and non-clinical groups. Results showed that there was a significant difference between the two groups for all three variables (*p* < 0.001; Table 1). Specific results indicated that the clinical group scored significantly higher than the non-clinical group on the CTQ [*t* = 4.84, *p* < 0.001, *g* = 0.59, 95% CI (0.33, 0.85)], the EMS [*t* = 8.13, *p* < 0.001, *g* = 0.98, 95% CI (0.71, 1.26)], and the TAS [*t* = 9.09, *p* < 0.001, *g* = 1.10, 95% CI (0.81, 1.38)], with all effect sizes falling in the moderate to large range (Table 1).

**Table 1:** Differences in the sores of CTQ, EMS and TAS between clinical and non-clinical groups.

	Clinical (*n* = 137)	Non-clinical (*n* = 134)	*t*	*p*	*g*	95% CI
CTQ	1.74 ± 0.47	1.49 ± 0.36	4.84	<0.001^***^	0.59	[0.33, 0.85]
EMS	2.73 ± 0.89	2.02 ± 0.49	8.13	<0.001^***^	0.98	[0.71, 1.26]
TAS	2.84 ± 0.63	2.24 ± 0.43	9.09	<0.001^***^	1.10	[0.81, 1.38]

*p<0.05, **p<0.01, ***p<0.001; CTQ: the score of childhood trauma; EMS: the score of early maladaptive schemas; TAS: the score of alexithymia.

Pearson correlation analysis was conducted to examine the relationships among childhood trauma, early maladaptive schemas, and alexithymia. The results revealed significant positive correlations between: childhood trauma and alexithymia (*r* = 0.172, *p* < 0.05); childhood trauma and early maladaptive schemas (*r* = 0.373, *p* < 0.05); and early maladaptive schemas and alexithymia (*r* = 0.653, *p* < 0.05). Detailed results are presented in Table 2.

**Table 2:** Pearson correlation between CTQ, EMS and TAS (*n* = 271).

	CTQ	EMS	TAS
CTQ	1.000		
EMS	0.373**	1.000	
TAS	0.172**	0.653**	1.000

### 3.3 Confirmatory factor analysis

To ensure the appropriate selection of observed variables for latent constructs and to prevent potential model non-convergence, confirmatory factor analysis (CFA) was first conducted for the observed variables of childhood trauma, early maladaptive schemas, and alexithymia prior to constructing the structural equation model. The acceptable model fit criteria in this study were as follows: the Comparative Fit Index (CFI) and Tucker-Lewis Index (TLI) should be greater than 0.90, and the Root Mean Square Error of Approximation (RMSEA) and Standardized Root Mean Square Residual (SRMR) should be less than or equal to 0.08 (Jiang and Liang, 2021). The analysis results indicated acceptable model fit indices. Specifically, for childhood trauma: *χ*^2^ = 433.403, df = 201, *p* < 0.01, CFI = 0.904, TLI = 0.9, RMSEA = 0.066, SRMR = 0.071; for early maladaptive schemas: *χ*^2^ = 15.163, df = 3, *p* < 0.01, CFI = 0.988, TLI = 0.959, RMSEA = 0.08, SRMR = 0.015; and for alexithymia: *χ*^2^ = 210.265, df = 74, *p* < 0.01, CFI = 0.919, TLI = 0.901, RMSEA = 0.08, SRMR = 0.066.

### 3.4 Mediation analysis of EMS

To further examine the underlying mechanism of alexithymia, a structural equation model (SEM) was constructed with early maladaptive schemas posited as a mediator. In the preliminary analysis, we included singleton status, gender, age, and educational level as covariates to control for their potential effects. Statistical tests indicated that neither singleton status nor gender significantly predicted the dependent variable (*p* > 0.05). Following the principle of model parsimony and to enhance statistical power, these two non-significant covariates were removed from the final model. Only age and educational level were retained as control variables for subsequent analyses. SEM was conducted using the maximum likelihood estimation method. Prior to estimating the structural model, confirmatory factor analyses (CFAs) were performed to validate the measurement models for each latent variable. Fit Indices for the Initial Mode:*χ*^2^/df = 3.258, GFI = 0.882, TLI = 0.881, CFI = 0.905, RMSEA = 0.092, 95% CI: [0.08,0.10]. The variance inflation factor (VIF) was employed to evaluate multicollinearity. The results showed VIF = 1 for all independent variables, confirming the absence of multicollinearity (O’Brien, 2007).

**Figure 1:**
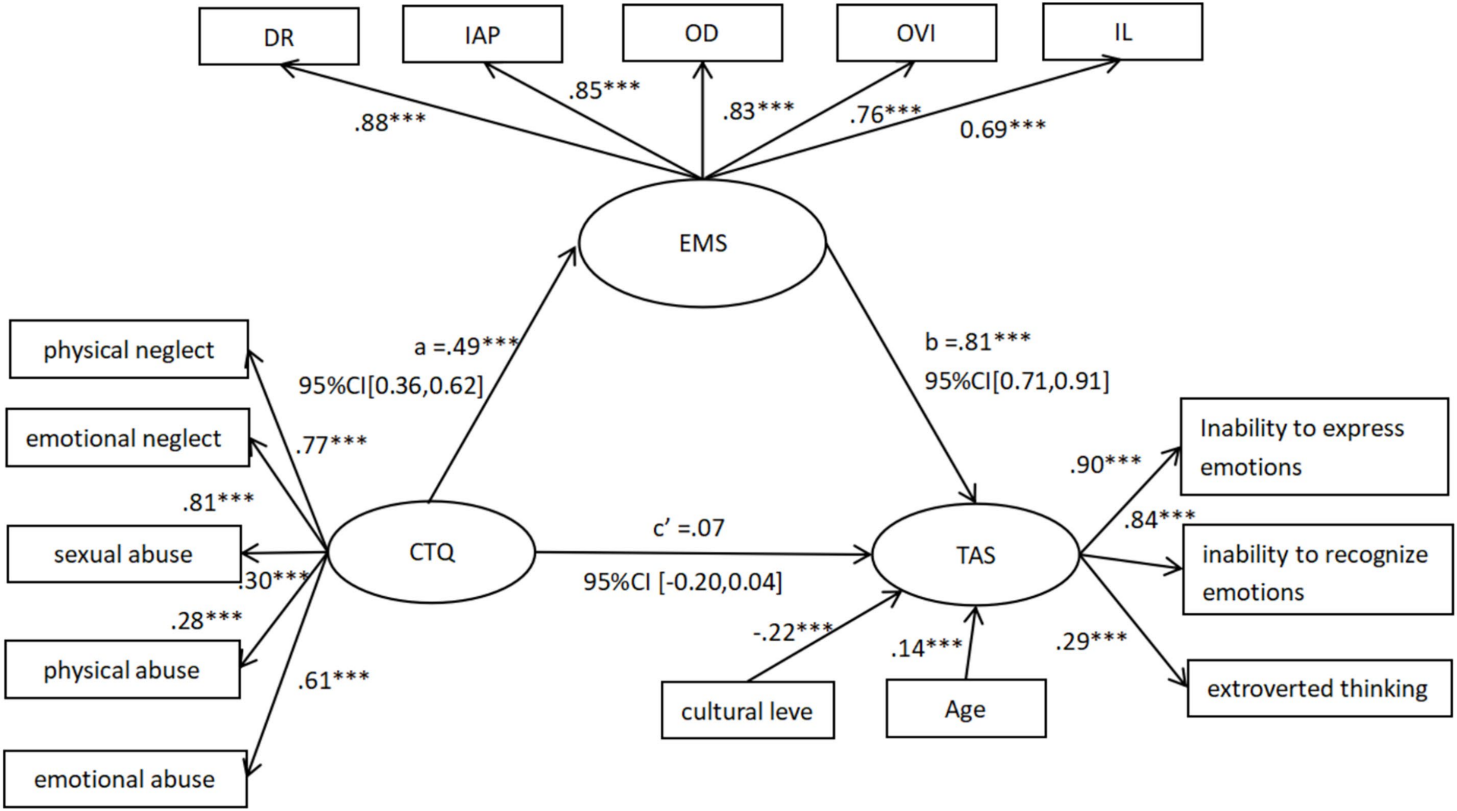
Mediation analysis of EMS (*N* = 270).

To test whether EMS mediate the relationship between childhood trauma and alexithymia, a bootstrapping procedure with 2000 resamples was employed to calculate bias-corrected 95% confidence intervals (CI). The indirect effect was considered statistically significant if the CI did not include zero. The results indicated that EMS fully mediated the relationship between childhood trauma and alexithymia, with a bias-corrected 95% CI of [0.29, 0.54], which did not include zero. Thus, EMS demonstrated a significant mediating effect between childhood trauma and alexithymia. Detailed results are presented in Figure 1 and Table 3.

**Table 3:** Mediation effects analysis.

Indirect effect	SE	95% CI	*p*
CTQ → EMS	0.067	[0.36,0.62]	0.001
EMS → TAS	0.050	[0.71,0.91]	0.001
CTQ → TAS	0.060	[−0.20,0.04]	0.199
CTQ → EMS → TAS	0.065	[0.29,0.54]	0.001

### 3.5 Multi-group path analysis

Prior to comparing the structural paths, measurement invariance was tested across the two groups. Given that both samples exceeded 100 participants, we sequentially constrained the factor loadings, intercepts, and residuals to test for configural, metric, scalar, and strict invariance (Cheung and Rensvold, 2002). The results indicated that the measurement model demonstrated acceptable metric invariance between the non-clinical and clinical groups (ΔCFI = 0.018 > 0.01, ΔRMSEA = 0.003 < 0.015), suggesting that the assumption of equal factor loadings was reasonably met. However, the criteria for scalar and strict invariance were not satisfied, as reflected by substantial deterioration in model fit (ΔCFI > 0.01). Based on the established metric invariance, we further examined structural path invariance by constraining the path coefficients to be equal across groups. The comparison results showed that imposing equality constraints on structural paths did not significantly degrade model fit (Δ*χ*^2^ = 6.345, *p* = 0.096; ΔCFI = 0.002; ΔRMSEA = 0.000). This finding indicates that the constrained model is acceptable, implying that the structural paths are statistically equivalent between the two groups. The establishment of metric invariance is generally regarded as sufficient for comparing structural relationships across groups, as recommended in the structural equation modeling (SEM) literature (Cheung and Rensvold, 2002). Therefore, under the condition of metric invariance, no significant structural differences were observed between the non-clinical and clinical groups. The results of the equivalence test are presented in Table 4.

**Table 4:** Equivalence testing between the non-clinical and the clinical groups.

Equivalence testing model	*χ*^2^(df)	CFI	ΔCFI	RMSEA	ΔRMSEA	Discrimination
Configural invariance	329.966 (168)	0.898	—	0.060		Invariance
Weak invariance	368.108 (178)	0.880	0.018	0.063	0.003	Invariance
Strong invariance	468.149 (191)	0.826	0.054	0.074	0.011	Non-invariance
Strict invariance	602.725 (207)	0.751	0.075	0.084	0.010	Non-invariance

To examine the group differences in structural paths between the non-clinical and clinical groups, a multi-group analysis was conducted. The results showed that only the path coefficients of CTQ → OVI, OVI → TES were significant in the clinical group (*p* < 0.01) and the path coefficients of CTQ → IL were significant (*p* < 0.01) and not significant in the non-clinical group (*p* > 0.05). The path coefficients of CTQ → OVI, OVI → TAS were significant (*p* < 0.01) in the clinical group, and CTQ → IL were significant (*p* < 0.01) in the non-clinical group. Multi-group analysis revealed that the path coefficients for CTQ → DR (*t* = 2.359, *p* < 0.05), CTQ → OVI (*t* = 2.601, *p* < 0.05), and OVI → TES (*t* = 2.288, *p* < 0.05) significantly differed between the clinical and non-clinical groups. Detailed results are presented in Table 5.

**Table 5:** Multi-group path analysis.

	Clinical group		Non-clinical group		Δ*χ*^2^	*p*
Path	*β*	*b*	*β*	*b*		
CTQ → DR	0.462^***^	1.256^***^	0.273^**^	0.487^**^	8.356	0.039
DR → TAS	0.681^***^	0.504^***^	0.595^***^	0.576^***^		
CTQ → TAS	0.074	0.149	0.051	0.087		
CTQ → IAP	0.317^**^	0.771^**^	0.289^***^	0.464^***^	1.340	0.720
IAP → TAS	0.665^***^	0.555^***^	0.497^***^	0.541^***^		
CTQ → TAS	0.011	0.023	0.066	0.115		
CTQ → OD	0.267^**^	0.685^**^	0.230^**^	0.458^**^	2.430	0.488
OD → TAS CTQ → TAS	0.563^***^	0.444^***^	0.358^**^	0.306^**^ 0.230		
	0.063	0.127	0.135			
CTQ → OVI	0.473^***^	1.254^***^	0.101	0.273	13.545	0.004
OVI → TAS	0.530^***^	0.380^***^	0.301	0.182		
CTQ → TAS	0.034	0.064	0.183	0.300		
CTQ → IL	0.277^**^	0.704^**^	0.161	0.372	1.615	0.656
IL → TAS CTQ → TAS	0.471^***^	0.389^***^	0.404^***^	0.320^***^		
	0.086	0.180	0.140	0.256		

## 4 Discussion

Chronic exposure to traumatic environments during childhood can engender profound negative emotions and insecurities, leading individuals to perceive the world as inherently dangerous (Snyder et al., 2024). Persistent physical and emotional neglect, coupled with inconsistent care, can result in unmet emotional needs, fostering the development of the disconnection and rejection, autonomy and incapacity domains of Early Maladaptive Schemas (EMS) (Wang et al., 2023). These schemas precipitate maladaptive coping strategies, whereby individuals become convinced that their core emotional needs cannot be met in relationships, subsequently impairing their ability to express emotions and needs, which contributes to elevated levels of alexithymia (May et al., 2022). The present study found that the disconnection and rejection schema domain significantly mediated the relationship between childhood trauma and alexithymia in the non-clinical sample. Specifically, experiences such as verbal, physical, or emotional abuse may foster schemas such as distrust/abuse and abandonment/instability, which are associated with alexithymia (Lim and Barlas, 2019). Consistent with recent findings, Feyzioğlu et al. (2022) investigated 435 healthy individuals (excluding those diagnosed with psychological disorders or using psychiatric medications) and demonstrated that childhood trauma serves as a predictive factor for alexithymia. When mediating variables were introduced to examine the indirect effects of childhood trauma on alexithymia, all five schema domains were found to significantly mediate this relationship. Specifically, caregiver abuse and neglect were associated with the development of the Disconnection and Rejection schema domain. Within this schema domain, individuals come to believe that their needs for belonging, love, care, safety, and stability cannot be met in any relationship, which predisposes them to higher levels of alexithymia (Bach et al., 2018). This is consistent with recent research emphasizing the role of EMS as a critical mechanism linking early adversity to emotional processing deficits, including alexithymia, across diverse populations (Tsouvelas et al., 2023).

Using multi-group path analysis, this study examined differences in the mediation model between clinical (depressed) and non-clinical (healthy) groups. Results revealed that the disconnection and rejection, impaired autonomy and performance, other-directedness, and impaired limits schema domains significantly mediated the relationship between childhood trauma and alexithymia in both samples. Therefore, regardless of whether an individual is diagnosed with depression, traumatic experiences in childhood may contribute to the formation of maladaptive schemas, thereby leading to the development of alexithymia. These findings are consistent with prior research indicating that alexithymia is not merely a symptom of depression or anxiety but a stable trait that increases vulnerability to psychiatric disorders (Kajanoja et al., 2021). Moreover, they align with longitudinal evidence highlighting the enduring and trans-diagnostic nature of the pathway from childhood trauma to alexithymia and subsequent mental distress (Anagnostopoulou et al., 2024).

These findings suggest that in the clinical sample, childhood trauma exhibits a more potent effect in activating both the disconnection and rejection and over-vigilance and inhibition schema domains. Notably, multi-group analyses highlighted a stronger association between the over-vigilance and inhibition schema domains and alexithymia in the clinical sample compared to the non-clinical sample.

This finding extends beyond mere group mean differences and points to qualitatively distinct psychopathological processes operating in depression. The more pronounced mediating role of the disconnection and rejection schema domain in the clinical group may reflect the interpersonal and attachment related complexities often comorbid with depression. Childhood trauma involving abuse or neglect can shape the disconnection and rejection schema domain, which encompasses an individual’s mistrust of others and anticipation of relational harm. In depression, these schemas may interact with social withdrawal and anhedonia, further undermining the motivation for emotional communication and exacerbating feelings of isolation key contributors to alexithymia. It is plausible that the clinical sample includes a subgroup with more severe relational trauma or underlying personality pathology, for whom the disconnection and rejection pathway is particularly salient. The potentiated role of the over-vigilance and inhibition schema domain aligns with cognitive models of depression that emphasize experiential avoidance and cognitive fusion. Individuals with depression often exhibit heightened sensitivity to negative internal states coupled with a propensity to suppress or ruminate on these experiences—a process directly mirrored in the over-vigilance and inhibition schema domain (Roelofs et al., 2016). Within the clinical context, these schemas may not only represent cognitive content but also active regulatory strategies that perpetuate alexithymia by chronically diverting attention away from emotional experience and toward cognitive control or performance standards. This creates a vicious cycle where emotional suppression is reinforced by schema driven beliefs about the danger or unacceptability of emotional expression. Notably, the lack of significant group differences in pathways through impaired autonomy, other-directedness, and impaired limits suggests these schemas may operate as more generalized trans-diagnostic vulnerability factors. They likely contribute to alexithymia through mechanisms such as low self-efficacy, excessive focus on others’ needs at the expense of self-awareness, or poor impulse and boundary regulation, which may be less specific to the depressive phenotype.

Previous studies have found that individuals in the depression group tend to exhibit relatively higher levels in the over-vigilance and inhibition schema domain, which exerts a persistent impact on the individual (Yarrington et al., 2023). Based on psychopathological research on depression, among individuals with comorbid depression and alexithymia, emotional distress may arise not only from cognitive difficulties in identifying feelings but also from a fear of emotional experiences and active suppression of such experiences, leading to heightened vigilance toward negative life events (Terock et al., 2020). From a psychopathological perspective, this aligns with the models of cognitive rumination and experiential avoidance observed in individuals with depression. Patients may exhibit hypervigilance toward internal emotional signals, perceiving them as threatening and uncontrollable. Consequently, they engage in emotional suppression in an attempt to regulate these experiences. However, such suppression may paradoxically lead to the accumulation of emotional distress, anhedonia, and increased cognitive load, thereby perpetuating a maladaptive cycle (Hessler and Everaert, 2023). Schemas such as negativity/pessimism, emotional inhibition, unrelenting standards, and punitiveness fall within this domain, reflecting a pervasive preoccupation with negative aspects of life (e.g., loss, guilt, betrayal) (Aydin and Kaya, 2024). Individuals with these schemas often prioritize perfectionistic standards over personal well-being, limiting emotional expression and interpersonal interactions, which further exacerbates alexithymia. The childhood environments of such individuals are often repressive, cold, and demanding, fostering hypervigilance toward negative events and negative emotionality, such as pessimism. This, in turn, impairs emotional regulation, paving the way for alexithymia (Mahta and Saeed, 2022; Bishop et al., 2022).

This study underscores the role of EMS as a cognitive vulnerability factor that may elevate the risk of psychiatric disorders, particularly among individuals with a history of childhood trauma. For healthy adults, early intervention targeting the effects of childhood trauma and maladaptive schemas could mitigate the development of alexithymia and associated psychological issues. Clinicians should prioritize assessing and addressing EMS to enhance emotional regulation and reduce vulnerability to affective disorders.

## 5 Implications

The study provides an etiological model of alexithymia within the framework of schema theory. Based on this model, clinicians treating depression should routinely assess not only standard symptoms but also childhood trauma, global alexithymia, and specific schema domains, with particular attention to the over-vigilance and inhibition and disconnection and rejection domains. The standardized YSQ can provide a detailed profile of cognitive vulnerabilities. When the over-vigilance and inhibition schema domain predominates in an individual, it alerts the clinician to potential issues such as emotional avoidance, perfectionism, and cognitive over-control. This may necessitate the integration of techniques beyond traditional cognitive restructuring, including mindfulness practices, acceptance-based strategies, and targeted emotion exposure exercises. When the over-vigilance and inhibition schema domain predominates in an individual, it alerts the clinician to potential issues such as emotional avoidance, perfectionism, and cognitive over-control. This may necessitate the integration of techniques beyond traditional cognitive restructuring, including mindfulness practices, acceptance and commitment therapy (ACT), and targeted emotion exposure exercises. For patients with a core disconnection and rejection schema, initial therapeutic efforts should be concentrated on establishing safety and trust within the therapeutic relationship. Schema therapy techniques, such as limited reparenting, can assist in addressing the unmet core needs for security and connection. Subsequent treatment can gently encourage appropriate emotional expression within this secure environment by employing Emotion-Focused Therapy (EFT) techniques. The trans-diagnostic role of other schemas suggests that interventions targeting self-efficacy, a healthy sense of self, and balanced attention to one’s own needs offer broad utility in reducing alexithymic traits across diverse clinical presentations.

## 6 Limitations and future research directions

For starters,the data in this research were primarily collected through subjective retrospective questionnaires, which assessed individuals’ recollections of childhood trauma. Participants may have experienced memory lapses, avoidance, distortions, or acceptance biases, potentially leading to discrepancies between the collected data and reality. Future studies could incorporate methods such as interviews to enhance data collection. A portion of the participants in this study were recruited from adult populations in hospital settings, which may affect the generalizability of the findings. This study is a cross-sectional investigation. Although it demonstrates the correlations between childhood trauma, early maladaptive schemas, and various dimensions of alexithymia, the limitations of the research design preclude the establishment of causal relationships. Future longitudinal studies could be conducted to explore the causal links among these three variables as well as their connections with the disease. An important limitation of this study is that the patient group with depression was not further stratified based on symptomatic presentations, severity levels, comorbid conditions, or treatment stages. Given the substantial heterogeneity of depression itself, our sample likely comprised individuals with various subtypes or comorbid conditions. Such heterogeneity could have confounded the observed strength of the mediating pathways, particularly the prominent role of the over-vigilance and inhibition schema domain in the clinical group. For example, the enhanced mediation via this pathway may be partly driven by the presence of individuals with comorbid anxiety or trauma-related disorders in the sample. Therefore, future studies are needed to examine the differential validity of this model across distinct subtypes of depression and various comorbid states (depression alone vs. depression with comorbid anxiety) through larger, prospectively stratified samples, in order to establish more targeted psychopathological models and intervention targets.

## 7 Conclusion

This study draws the following conclusions: First, patients with depression exhibit higher levels of childhood trauma, early maladaptive schemas, and alexithymia compared to healthy individuals. Second, the early maladaptive schema domains—disconnection and rejection,impaired autonomy and performance, other-directedness, over-vigilance and inhibition, and impaired limits—fully mediate the relationship between childhood trauma and alexithymia in depressive patients. Third, compared to the healthy group, the association between the over-vigilance and inhibition schema domain and alexithymia is stronger in patients with depression.

## Data Availability

The datasets presented in this study can be found in online repositories. The names of the repository/repositories and accession number(s) can be found in the article/supplementary material.
